# Class I and II Histone Deacetylase Inhibitors as Therapeutic Modulators of Dilated Cardiac Tissue-Derived Mesenchymal Stem/Stromal Cells

**DOI:** 10.3390/ijms25126758

**Published:** 2024-06-19

**Authors:** Rokas Mikšiūnas, Siegfried Labeit, Daiva Bironaite

**Affiliations:** 1 Department of Regenerative Medicine, State Research Institute Centre for Innovative Medicine, Santariškių 5, LT-08406 Vilnius, Lithuania; rokas.miksiunas@gmail.com; 2 Myomedix Ltd., Im Biengarten 36, 69151 Neckargemuend, Germany; labeit@medma.de

**Keywords:** dilated cardiomyopathy, histone deacetylate inhibitors, mesenchymal stem/stromal cells

## Abstract

The prevalence of dilated cardiomyopathy (DCM) is increasing globally, highlighting the need for innovative therapeutic approaches to prevent its onset. In this study, we examined the energetic and epigenetic distinctions between dilated and non-dilated human myocardium-derived mesenchymal stem/stromal cells (hmMSCs) and assessed the effects of class I and II HDAC inhibitors (HDACi) on these cells and their cardiomyogenic differentiation. Cells were isolated from myocardium biopsies using explant outgrowth methods. Mitochondrial and histone deacetylase activities, ATP levels, cardiac transcription factors, and structural proteins were assessed using flow cytometry, PCR, chemiluminescence, Western blotting, and immunohistochemistry. The data suggest that the tested HDAC inhibitors improved acetylation and enhanced the energetic status of both types of cells, with significant effects observed in dilated myocardium-derived hmMSCs. Additionally, the HDAC inhibitors activated the cardiac transcription factors Nkx2-5, HOPX, GATA4, and Mef2C, and upregulated structural proteins such as cardiac troponin T and alpha cardiac actin at both the protein and gene levels. In conclusion, our findings suggest that HDACi may serve as potential modulators of the energetic status and cardiomyogenic differentiation of human heart hmMSCs. This avenue of exploration could broaden the search for novel therapeutic interventions for dilated cardiomyopathy, ultimately leading to improvements in heart function.

## 1. Introduction

Cardiovascular malfunction is among the most prevalent heart issues, leading to over a million hospitalizations annually in the United States and Europe [1]. Individuals diagnosed with dilated cardiomyopathy (DCM) exhibit a reduced thickness of the heart wall structure, an increased diameter of the left ventricle chamber, and systolic dysfunction [2]. The etiology of DCM is often complex, involving various factors such as a toxic environment, inflammation, autoimmune or viral diseases, and other elements that impair blood supply and heart function, ultimately leading to heart failure and the need for transplantation [3]. At the tissue level, dilated hearts manifest multiple abnormalities associated with altered biological and physiological heart parameters, including reduced ejection fraction and impaired structural (sarcomere) and cellular functioning properties [4,5]. Diagnosing and treating cardiomyopathy is a challenging task due to the prolonged duration of the disease, the late onset of symptoms, and the impairment of multiple intracellular and extracellular molecular mechanisms [6]. Consequently, there is an ongoing need to explore new therapeutic approaches to enhance the functioning of human dilated myocardium.

One potential method for modifying heart tissue involves the epigenetic modification of human mesenchymal stem/stromal cells (hmMSC) by altering protein acetylation and regulating deacetylase activity. Histone deacetylases (HDACs) play a crucial role in catalyzing the removal of acetyl groups from target proteins and are categorized into several distinct classes. Class I HDACs (HDAC 1, 2, 3, 8) are widely expressed throughout the entire organism, while class IIa (HDAC 4, 5, 7, 9) and IIb (HDAC 6, 10) are more specific to certain tissues. In terms of cardiac pathology, both classes can contribute to the development of heart disease. For instance, the overexpression of class I HDACs, including HDAC1 or HDAC2 in rat cardiac hypertrophy models in vivo, has been shown to promote fibrosis, inflammation, and ultimately heart failure [7,8]. Additionally, class II HDACs are relevant for heart function; for example, overexpression of *HDAC4* or excessive activity of HDAC6 has been shown to increase vulnerability to ischemia/perfusion injury in in vivo rat and mouse models [9,10].

HDAC inhibitors are commonly employed in cancer treatment; however, a growing body of data also show their anti-inflammatory effects, which involve reducing HDAC activity and even promoting oxidative phosphorylation [11,12,13]. Furthermore, universal HDAC inhibitors such as suberoylanilide hydroxamic acid (SAHA) or Trichostatin A (TSA) have been shown to mitigate fibrotic processes and enhance lung compliance in a mouse model of bleomycin-induced pulmonary fibrosis, improve liver hepatic function in a rat bile duct ligation model, and reduce cardiac hypertrophy in a mouse pressure overload model [14,15,16]. Mocetinostat, a class I HDAC inhibitor, has been shown to reverse cardiac fibrosis and improve heart function in post-myocardial infarction rats, while class II HDAC inhibitors like MC1568 have been studied in the context of heart failure [8]. Therefore, the inhibition of either class I or II HDACs, or both, by different HDAC inhibitors may exert distinct effects on heart tissue regeneration.

The heart consumes vast amounts of energy, and must continuously produce large amounts of ATP to sustain contractile function [17]. Mitochondrial oxidative phosphorylation normally contributes ~95% of all myocardial ATP requirements, while glycolysis provides the remaining 5% [18]. Therefore, cardiac energetic impairments, particularly mitochondrial, can cause various inherited and acquired heart diseases. For example, patients suffering from heart failure often show impaired mitochondrial function and reduced oxidative phosphorylation [19,20,21]. The role of mitochondria is also important in the pathophysiology of atherosclerosis, ischemia–reperfusion injury, hypertension, diabetes, cardiac hypertrophy and others due to the uncontrolled production of reactive oxygen species (ROS) [22]. On the other hand, a certain level of ROS is essential for cardiovascular function at the physiological level, including calcium signaling, cell cycle control and differentiation [23]. Therefore, all effectors positively affecting the energetic status of heart cells may also positively affect the functioning of human DCM hearts.

So far, most studies on HDAC inhibitors have focused primarily on in vivo models, leaving the relationship between the HDAC activity, cellular energetic status, and cardiomyogenic differentiation of DCM heart-derived hmMSCs compared to healthy tissue cells largely unexplored. Therefore, the main aim of this study was to investigate the effects of class I and II HDAC inhibitors (SAHA, MC1568, and mocetinostat) on the intracellular processes regulating cardiomyogenic differentiation of healthy and pathological/dilated myocardium-derived hmMSCs. Investigating the molecular mechanisms of epigenetic HDAC regulators at the cellular level allows for further extension of their therapeutic application for the prevention or mitigation of DCM effects in human hearts.

## 2. Results

### 2.1. Isolation and Characterization of Human Myocardium-Derived Primary Mesenchymal Stem/Stromal Cells

**Figure 1:**
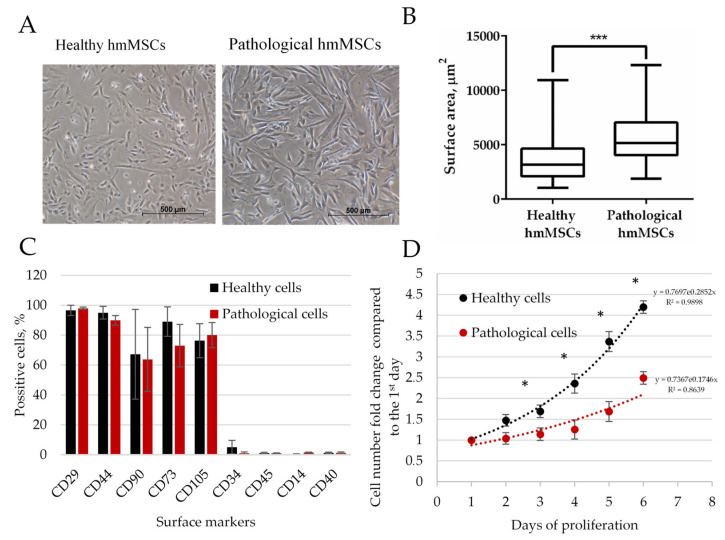
Molecular characterization of non-dilated/healthy and dilated/pathological myocardia-derived hmMSCs. (**A**) Morphology of healthy and pathological hmMSCs. (**B**) Distribution of healthy and pathological cells according to the cell attachment area calculated from 100 attached cells. (**C**) Surface marker profile in healthy and pathological hmMSCs. (**D**) The proliferation rate of healthy and pathological hmMSCs. Data are statistically significant at * *p* < 0.05 and *** *p* < 0.01, n = 3, comparing healthy and pathological cells.

Human myocardium-derived MSCs were successfully isolated using the explant outgrowth method from both dilated/DCM/pathological and non-dilated/healthy heart myocardia (Figure 1A). Both healthy and pathological myocardium-derived cells exhibited spindle-like morphology, although healthy hmMSCs were slightly smaller in size and grew more compactly than pathological ones. Size measurements of more than 100 cells revealed that non-dilated/healthy hmMSCs were almost two-fold smaller than pathological ones (Figure 1B). Both types of isolated hmMSCs were positive for CD29, CD44, CD90, CD73, and CD105, and negative for CD34, CD14, CD45, and CD40, which are classic MSC surface markers (Figure 1C). Proliferation rate measurements, conducted using both CCK-8 and cell counting methods, also confirmed that non-dilated myocardium/healthy hmMSCs proliferated almost two-fold faster than pathological/dilated heart-derived hmMSCs (Figure 1D).

### 2.2. The HDAC Activity and Expression Level in Both Cell Types and the Effect of HDACi

**Figure 2:**
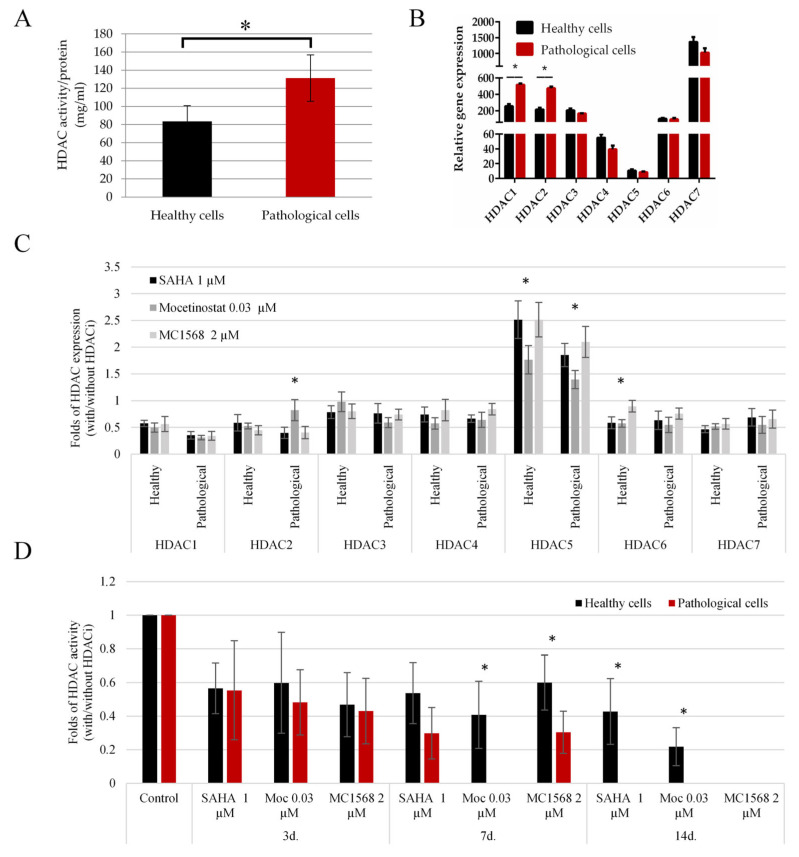
HDAC activity and expression profiles in healthy and pathological hmMSCs. (**A**) HDAC activity of healthy and pathological hmMSCs (arbitrary fluorescence units/protein (mg/mL)). (**B**) Relative expression of HDAC 1–7 (2^−ΔCT^) of healthy and pathological hmMSCs. (**C**) Relative expression of HDAC 1–7 genes in healthy and pathological hmMSCs after the exposure to SAHA (1 µM), mocetinostat (0.03 µM) and MC1568 (2 µM) for 3 days. (**D**) Change of HDAC activity in healthy and pathological cells after exposure to SAHA (1 µM), mocetinostat (0.03 µM) and MC1568 (2 µM) for 3, 7, 14 days; ratio to the control cells. Data are statistically significant at * *p* < 0.05, n = 3, comparing healthy and pathological cells (**A**–**C**) and effects of inhibitors on those cells (**D**).

To assess HDAC activity, we utilized the fluorescent HDAC substrate BOC-Ac-Lys-AMC. Pathological cells exhibited 1.5-fold greater HDAC activity compared to healthy hmMSCs (Figure 2A). Additionally, pathological myocardium-derived cells displayed a two-fold higher expression of HDAC1 and HDAC2 compared to healthy myocardium cells, while the expression levels of HDAC3, HDAC5, and HDAC6 were fairly similar between pathological and healthy hmMSCs (Figure 2B). Furthermore, the expression level of the HDAC7 gene was highest in both healthy (1362.8 ± 123.8) and pathological (1024.2 ± 112.2) cells, while the expression level of HDAC5 was lowest (healthy cells 10.5 ± 1.2, pathological cells 8.2 ± 0.8) in both types of cells.

In addition, all HDAC inhibitors suppressed the expression of HDAC1, HDAC4, HDAC3, HDAC2, HDAC7, and HDAC6 in both pathological and healthy hmMSCs after 3 days of incubation (Figure 2C). Despite the relatively low expression of HDAC5 in both types of cells compared to the other HDACs (Figure 2B), the addition of SAHA, mocetinostat, or MC1568 resulted in upregulated HDAC5 expression (Figure 2C). It is conceivable that the intracellular transcriptional regulation of HDAC genes involves a compensatory mechanism, leading to variable responses to HDAC inhibitors among different HDACs.

Finally, the suppression of HDAC activity by all HDAC inhibitors (1 µM SAHA, 0.03 µM mocetinostat, and 2 µM MC1568) was more pronounced in pathological cells than in healthy cells during longer incubation periods of 7 and 14 days (Figure 2D). These data suggest that pathological cells are more responsive to epigenetic regulation by HDAC inhibitors than healthy cells.

### 2.3. Energetic Status of Healthy and Pathological hmMSCs and the Effect of HDACi

**Figure 3:**
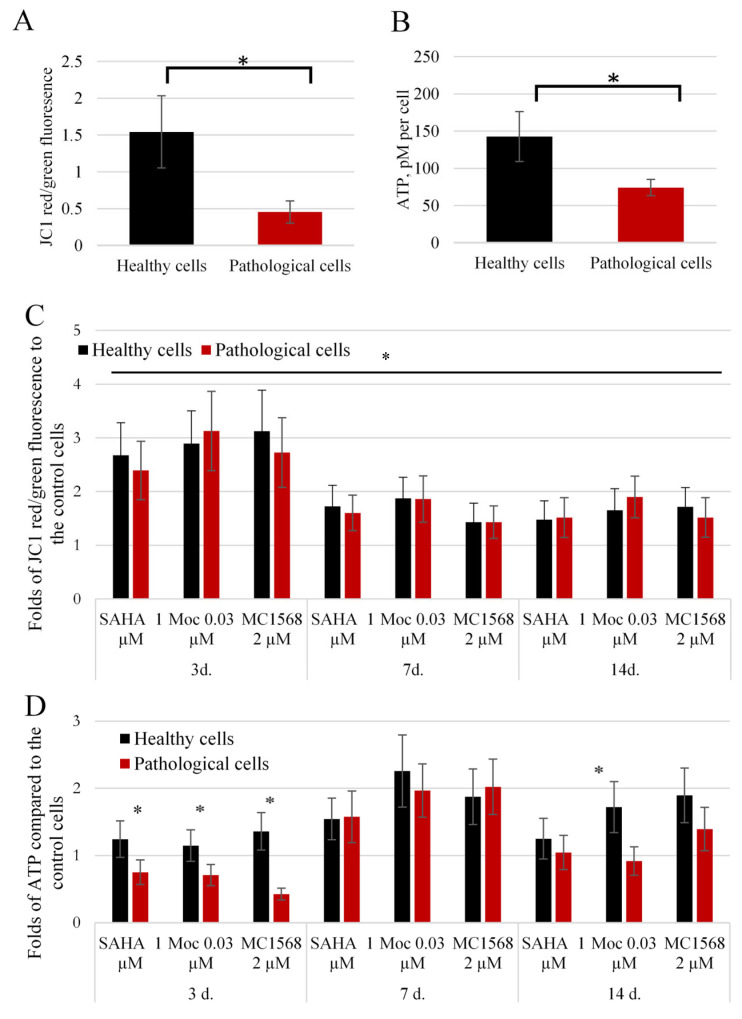
The energetic status of healthy and pathological hmMSCs and impact of HDAC inhibitors. (**A**) The mitochondrial membrane potential of healthy and pathological hmMSCs. (**B**) The total level of ATP per cell in healthy and pathological hmMSCs. (**C**,**D**) Folds of change of mitochondrial membrane potential and ATP production, respectively, in healthy and pathological hmMSCs after exposure to SAHA (1 µM), mocetinostat (0.03 µM) and MC1568 (2 µM) for 3, 7 and 14 days. Data are statistically significant at * *p* < 0.05, n = 3, comparing 3, 7 and 14 days (**C**) and healthy and pathological cells (**D**).

The protein acetylation process may influence multiple intracellular functions. Therefore, we examined whether the tested HDACi were capable of improving the energetic status of healthy and pathological hmMSCs. The data indicate that at the control level, the mitochondrial membrane potential (MMP), measured with JC1 (Figure 3A) and ATP levels (Figure 3B), were approximately three-fold higher in healthy hmMSCs compared to pathological hmMSCs. All HDAC inhibitors significantly increased MMP activity in both healthy and pathological hmMSCs during the first 3 days of incubation (Figure 3C), while the total ATP levels were upregulated only after 7 days of exposure to the HDAC inhibitors (Figure 3D). These findings suggest that MMPs responded more quickly to the HDAC inhibitors, whereas ATP production required a longer period. Healthy hmMSCs exhibited slightly better ATP generation compared to pathological cells.

### 2.4. The Effect of HDACi on ROS and Lysine Acetylation (Kac)

**Figure 4:**
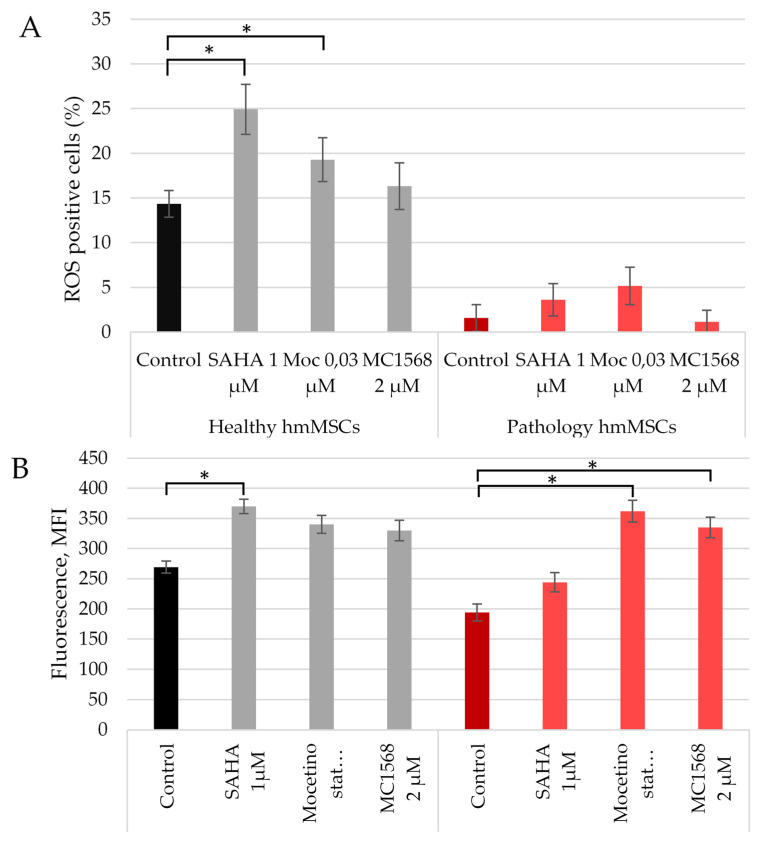
The effect of HDAC inhibitors on the intracellular ROS levels and total level of acetylated lysine in healthy and pathological cells. (**A**) The change of intracellular levels of ROS in healthy and pathological hmMSCs w/o and with HDACi. (**B**) The total level of acetylated proteins in both types of cells incubated with SAHA (1 µM), mocetinostat (0.03 µM) and MC1568 (2 µM) for 3 days. Data are statistically significant at * *p* < 0.05, n = 3, comparing the effects of HDAC inhibitors on healthy and pathological cells with control cells.

It is known that higher mitochondrial activity correlates with higher intracellular mitochondrial membrane potentials and elevated levels of intracellular reactive oxygen species (ROS). Given that HDAC inhibitors (HDACi) increased MMPs in both types of hmMSCs, it was intriguing to explore whether HDACi also augmented intracellular ROS levels and protein acetylation during an initial 3-day incubation period (Figure 4). The data reveal that at the control level, healthy cells exhibited a higher level of ROS compared to pathological cells, which was further increased by all HDACi (Figure 4A). Additionally, the HDAC inhibitors also elevated the total level of acetylated lysine in both types of hmMSCs (Figure 4B). These findings suggest that the activation of MMPs during 3-days of incubation with HDACi (Figure 3C) is associated with a non-cytotoxic increase in ROS and protein acetylation (Figure 4). A similar effect of ROS on protein acetylation was recently demonstrated in plants [24]. Further, more detailed investigations into the effects of ROS on protein acetylation in heart-derived hmMSCs are warranted.

### 2.5. The Effect of HDACi on Cardiomyogenic Differentiation Regulating Transcription Factors

**Figure 5:**
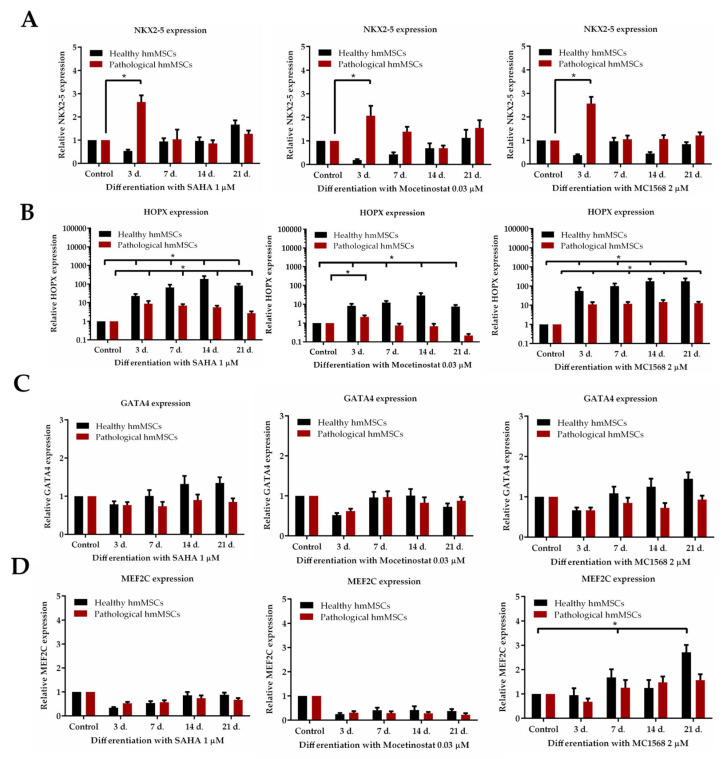
The impact of HDAC inhibitors on cardiac transcription factors in healthy and pathological cells. Folds of change of gene expression: (**A**) *NKX2-5*; (**B**) *HOPX*; (**C**) *GATA4*; and (**D**) *MEF2C*, (2^−ΔΔCT^) during 21 days of exposure to SAHA (1 µM), mocetinostat (0.03 µM) and MC1568 (2 µM) in healthy and pathological cells. * Data are statistically significant at *p* < 0.05, n = 3, comparing the effects of HDAC inhibitors on healthy and pathological cells with control cells.

**Figure 6:**
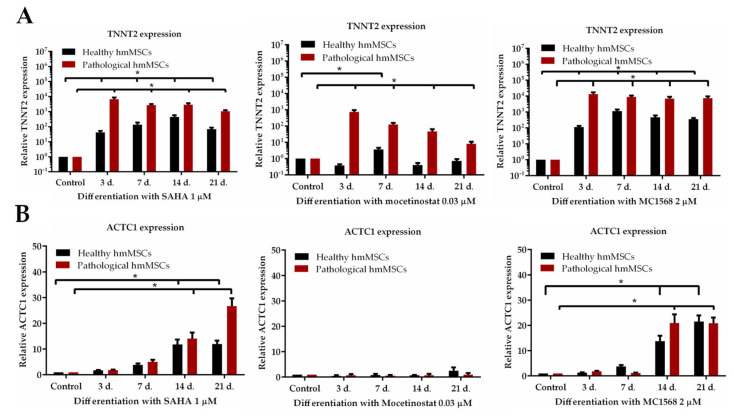
The impact of HDAC inhibitors on sarcomeric proteins of healthy and pathological cells. Folds of change of expression: (**A**) TNNT2; (**B**) ACTC1 genes (2^−ΔΔCT^) during 21 days of exposure to SAHA (1 µM), mocetinostat (0.03 µM) and MC1568 (2 µM) in healthy and pathological cells. * Data are statistically significant at *p* < 0.05, n = 3 comparing the effects of HDACi on healthy and pathological cells with control cells.

Finally, we investigated the effect of HDAC inhibitors on the expression of cardiac transcription factors *NKX2-5*, *HOPX*, *GATA4*, and *MEF2C* (Figure 5) and structural proteins cardiac troponin T and alpha actin (Figure 6) in both types of cells exposed to the HDAC inhibitors for 21 days.

All HDAC inhibitors, SAHA, mocetinostat, and MC1568, promoted significant increases in the expression of the main cardiomyogenic differentiation-regulating transcription factor *NKX2-5* in pathological cells (2.64 ± 0.24, 2.07 ± 0.4, and 2.57 ± 0.28), respectively, but not in healthy hmMSCs during the first 3 days of incubation (Figure 5A).

Similarly, all HDAC inhibitors showed an early but more prolonged expression of *HOPX* compared to *NKX2-5*, i.e., the 1 µM of SAHA and 2 µM of MC1568 were most effective on *HOPX* expression in healthy cells (82.7 ± 20.6 and 178.5 ± 73.2), compared to the pathological cells (2.7 ± 0.6 and 12.7 ± 2.6), at the 21st day of differentiation (Figure 5B).

SAHA and MC1568, but not mocetinostat, showed prolonged expression of *GATA4* in healthy cells, which was 1.35 ± 0.14 and 1.45 ± 0.15 fold higher than in pathological cells, at the 21st day of differentiation (Figure 5C).

Finally, only MC1568 showed upregulation of the transcription factor *MEF2C* in both types of cells, with a more significant effect on healthy cells at the 21st day of differentiation (Figure 5D).

Altogether, the data indicate that *NKX2-5* and *HOPX* may serve as early transcription factors regulating cardiomyogenic differentiation as they more quickly responded to the HDAC inhibitors within the first 3 days of incubation. Conversely, *GATA4* and *MEF2C* act as late transcription factors, becoming activated during the 21 days of exposure to the HDAC inhibitors.

### 2.6. The Effect of HDACi on Structural Cardiomyogenic Proteins

**Figure 7:**
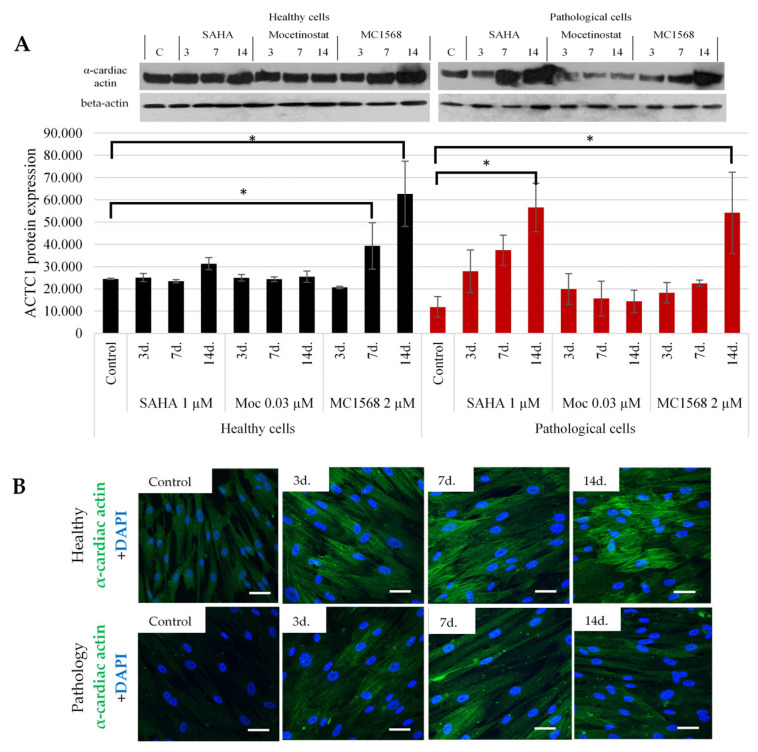
The impact of HDAC inhibitors on the levels of alpha cardiac actin in healthy and pathological hmMSCs. (**A**) The level of alpha cardiac actin in healthy and pathological hmMSCs after exposure to SAHA (1 µM), mocetinostat (0.03 µM), and MC1568 (2 µM) analyzed using Western blotting. Quantitative level of alpha cardiac actin was calculated using the ImageJ program and shown as staining intensity (arbitrary units) compared to the background. Data are shown as mean ± standard deviation (SD) and are significant comparing cells with/without HDACi at * *p* ≤ 0.05, n = 3 from two experiments calculated using the Excel program. (**B**) Confocal microscope micrographs of alpha cardiac actin in healthy and pathological hmMSCs after exposure to 1 µM of SAHA for 3 d., 7 d., and 14 d. Scale bar = 20 μm. Control—healthy and pathological hmMSCs without HDACi.

Next, it was beneficial to investigate the effect of HDAC inhibitors on the main cardiac structural proteins such as troponin T (*TNNT2*) and alpha cardiac actin (*ACTC1*) at both the gene and protein levels (Figure 6 and Figure 7). The pathological cells better responded to the HDAC inhibitors, SAHA, mocetinostat, and MC1568, and exhibited a stronger increase in *TNNT2* expression (6700 ± 2010, 739 ± 132, and 13,400 ± 2304, respectively) compared to the healthy cells (41 ± 13, 0.38 ± 0.15, and 114.56 ± 15, respectively) during the first 3 days of exposure (Figure 6A). SAHA and MC1568 exhibited a more stable *TNNT2* expression, compared to mocetinostat, during 21 days of exposure.

Similarly, the HDAC inhibitors SAHA and MC1568 were more effective in promoting the expression of another cardiac structural protein, alpha cardiac actin, in both healthy and pathological hmMSCs at the 21st day of exposure, i.e., 1 μM SAHA increased the expression of *ACTC1* by 11.9 ± 1.3 and 26.7 ± 4.3, while MC1568 increased it by 21.5 ± 2.3 and 20.8 ± 2.2 folds, respectively (Figure 6B). The effect of the HDAC inhibitors SAHA and MC1568 on the expression of alpha cardiac actin was more pronounced than that of mocetinostat.

To confirm the effect of HDAC inhibitors on the main component of thin myofibril filaments and alpha cardiac actin, we investigated the levels of actin using Western blotting (Figure 7A) and microscopically (Figure 7B) in both healthy and pathological hmMSCs with and without the HDAC inhibitors. The data confirm that the HDAC inhibitors SAHA and MC1568 increased the level of alpha cardiac actin and promoted the formation of cardiac tissue-typical fibers in both healthy and pathological cells. Both types of cells exhibited less responsiveness to mocetinostat, suggesting that its effect might be too strong to activate cardiac transcription factors and structural proteins at both the protein and gene levels.

## 3. Discussion

Idiopathic dilated cardiomyopathy (DCM) represents a prevalent condition responsible for approximately 50% of all heart failure cases. Beyond heart failure, DCM can precipitate arrhythmias and even sudden cardiac death at any age. Idiopathic DCM often exhibits familial or genetic predispositions, with single mutations primarily impacting tissue contraction-regulating proteins, resulting in ventricular chamber dilation and reduced systolic performance rather than hypertrophy [25,26]. Alongside genetic factors, viral, immune, and toxic etiologies, such as drugs, environmental toxins, and chemicals, warrant consideration [27]. DCM manifests as a chronic and enduring ailment typically diagnosed only after symptom onset. Consequently, there exists a critical necessity to scrutinize the molecular mechanisms underpinning DCM development to advance therapeutic and preventive strategies for this disorder. In this study, we explore the levels of various HDACs and energetic activities in myocardium-derived hmMSCs from both healthy and dilated hearts, alongside their regenerative potential modulated by HDAC class I and II inhibitors.

Evidence supporting the importance of epigenetic regulation in cardiac diseases is rapidly accumulating. However, the role of HDACs has predominantly been investigated in cardiac hypertrophy and heart failure [28,29]. Nonetheless, the impact of HDACs and their inhibitors on the development and/or treatment of DCM remains unclear. Studies have demonstrated that long-term inhibition of histone deacetylase with valproic acid attenuated hypertrophic and hypertensive responses by modulating reactive oxygen species and pro-inflammatory cytokines in spontaneously hypertensive rats [30]. HDAC inhibitors such as Trichostatin A, valproic acid, or SK-7041 have been shown to partially suppress Ang II or aortic banding-induced cardiac hypertrophy and improve mouse survival [14]. Additionally, the HDAC inhibitor MPT0E014 has been found to reduce cardiac hypertrophy and fibrosis, improve cardiac contractility, and attenuate structural remodeling in isoproterenol-induced mice with dilated cardiomyopathy [31]. In addition to the aforementioned mechanisms of action in the heart, HDAC inhibitors have also been shown to improve the energetic status of DCM heart tissue-derived MSCs in vitro [32]. The data from this study demonstrate that HDAC inhibitors SAHA, mocetinostat, and MC1568 increased MMP levels during the first 3 days of incubation, while ATP levels was increased after the 7 days of incubation in both cell types.

Furthermore, in this study, we observed dependencies between the upregulated levels of ROS, MMP activation, acetylation, and the expression of certain cardiac genes (*Nkx2-5*, *HOPX*, and *TNNT2*) during the initial 3 days of incubation with HDAC inhibitors. ROS are frequently associated with heart failure and cardiovascular pathology, including ischemic disease, cardiac hypertrophy, and dilated cardiomyopathy [23]. Excessive ROS production and oxidative stress impair cellular signaling and promote cardiomyocyte death [33]. However, it has been demonstrated that a certain low level of ROS is essential for cardiovascular functioning at a physiological level, including cell differentiation [23]. Evidence suggests the existence of an optimal, low level of ROS, referred to as the ‘redox window’ [34]. Similar observations have been made regarding mitochondrial ROS, which at physiological levels promote mitochondrial biogenesis, but at excessive levels induce pathological changes that damage mitochondrial DNA [35,36]. Studies have shown that C2C12 muscle skeletal cells stimulated with H2O2 in vitro promote the expression of PGC-1α [37], a target already considered beneficial for promoting mitochondrial biogenesis and activating the antioxidant system in heart failure patients [38]. Therefore, the slight increase in ROS in heart hmMSCs after exposure to HDAC inhibitors observed in this study might be indicative of mitochondrial activation and subsequent intracellular changes such as cardiomyogenic differentiation. However, this aspect requires further detailed investigation.

The functioning of the heart is regulated by a complex interplay of transcription factors, structural proteins involved in contraction, ion channels, and other factors. Among these, NK2 homeobox 5 (NKX2-5), a homeobox-containing transcription factor, stands out as a key regulator of cardiomyogenic differentiation. Mutations in NKX2-5 are associated with a spectrum of congenital heart diseases, including postnatal progressive conduction defects and occasional left ventricular dysfunction [39,40]. Studies on mice with perinatal loss of NKX2-5 have underscored its critical importance for cardiac conduction, contraction, particularly during the perinatal period [41], and hypertrophy [28]. Furthermore, direct interactions between various transcription factors, such as NKX2-5 and MEF2C (myocyte-specific enhancer-binding factor 2c), have been identified, mediating molecular programs that regulate mammalian heart development [42]. MEF2C, which specifically binds to the MEF2 element and activates the transcription process, can be regulated by stress-responsive kinases that specifically affect MEF2–HDAC interactions [43]. The data from this study indicate that *NKX2-5* in hmMSCs responded more strongly and quickly (within the first 3 days of incubation) to all HDAC inhibitors compared with *MEF-2C*. These findings suggest that MEF-2C may require a stronger stimulus than the HDAC inhibitors used in both cell types.

NKX2-5 functioning is closely connected to another homeobox only protein homeobox (HOPX), which contains a homeobox-like domain but lacks certain conserved residues required for DNA binding and functions downstream of NKX2-5 [44]. Moreover, it was shown that expression of the HOPX gene is initiated in early cardiogenesis, and continues in cardiomyocytes throughout embryonic and postnatal development [44]. Another study has indicated that HOPX activation is critical for regulating the late stages of cardiomyocyte maturation, suggesting that HOPX can act as a determinant and molecular switch between cardiomyocyte progenitor and gene maturation programs [45]. Moreover, there has been evidence of an interaction between GATA4 and HOPX, i.e., HDAC2 physically interacts with GATA4, and this interaction is stabilized by HOPX. In the absence of HOPX and HDAC2, hyperacetylation of GATA4 is associated with a significant increase in cardiac myocyte proliferation, upregulation of GATA4 target genes, and perinatal lethality of mouse embryos [46]. Our data indicate that *HOPX*, akin to *NKX2-5*, exhibits a quicker response to HDACi during the first 3 days of incubation compared to *GATA4* activation, with a more pronounced effect on healthy hmMSCs compared to pathological ones.

Finally, both in vitro and in vivo studies demonstrate the response of structural and heart contraction-regulating proteins to HDAC inhibitors. HDACi SAHA, for instance, was observed to elevate the levels of alpha-smooth muscle actin (α-SMA), cardiac muscle troponin T (*TNNT2*), desmin, and cardiac muscle alpha actin (*ACTC1*) in dental pulp-derived cells [47]. A recent publication also revealed that the HDAC inhibitor valproic acid upregulated the immune response gene 1 (IRG1) in pig hearts [48]. Similarly, another HDAC inhibitor, 4-phenylbutyrate (4-PBA), induced the early stages of cardiac differentiation regulators, transcription factors ISL1 and NKX2-5 in mouse embryonic cells, albeit with a reduction in the levels of structural protein cardiac troponin T [49]. The data from this study suggest that HDACi SAHA, mocetinostat, and MC1568, similar to transcription factors *NKX2-5* and *HOPX*, more effectively activate the expression of *TNNT2* during the initial 3 days of cell incubation, while *ACTC1* activation, similar to *GATA4* and *MEF2C*, requires a longer incubation period (14–21 days). It is possible that the rapid activation of cardiac genes by HDACi is linked to improved mitochondrial function and cellular energy status, initiating cardiomyogenic differentiation.

In summary, the data suggest that myocardium-derived hmMSCs from DCM hearts exhibited increased size, poorer proliferation, and lower mitochondrial activity compared to the healthy tissue hmMSCs. Class I and II HDAC inhibitors, including SAHA, mocetinostat, and MC1568, improved mitochondrial activity by increasing MMPs and the total level of ATP, while also stimulating cardiac differentiation-related transcription factors and structural proteins. These findings indicate that HDAC inhibitors merit further investigation not only as anti-cancer compounds but also as potential therapeutics for DCM, aiming to regenerate the functions of the dilated human heart.

## 4. Materials and Methods

### 4.1. Cell Isolation and Cultivation

Human heart biopsies were obtained from patients at the Santariškių clinic after signing informed consent forms (Bioethics No. 158200-14-741-257). Samples were prepared as written in [32]. Briefly, healthy and dilated human heart myocardium tissue biopsies were cut into 1–4 mm^3^ fragments, minced and washed in PBS with 2% PEST and partially digested with trypsin-EDTA solution at 37 °C for 10 min. Heart tissue explants were cultured in 6 well plates coated with 2 µg/mL of fibronectin in IMDM medium (Thermo Fisher Scientific, Waltham, MA, USA) supplemented with 20% FBS (Thermo Fisher Scientific, Waltham, MA, USA), 100 U/mL penicillin G, and 100 U/mL streptomycin (Thermo Fisher Scientific, Waltham, MA, USA) at 37 °C and 5% CO_2_. After 1–4 weeks, round bright cells migrated out of the tissue and attached to the plastic surface. When cardiac outgrowth reached 80% confluence, cells were lifted with trypsin and seeded for further experiments or frozen for storage.

### 4.2. Flow Cytometry Analysis of Cell Surface and Intracellular Markers

For the estimation of cell surface biomarkers, the cultivated cells were trypsinized, washed with PBS and blocked with PBS and 1% BSA solution at RT for 1 h to inhibit non-specific interactions. After the blocking, cells were washed and incubated for a half an hour on ice with specific antibodies or isotype controls: cluster of differentiation cell–cell adhesion factor (CD34-ImmunoglobulinG1-Fluorescein isothiocyanate (1F-297-T100, Exbio, Praha, Czech Republic)), costimulatory protein found on antigen-presenting cells (CD40-ImmunoglobulinG1-Allophycocyanin (555591, BD Biosciences, San Jose, CA, USA)), ecto-5′-nucleotidase (CD73-ImmunoglobulinG1-Fluorescein isothiocyanate (561254, BD Biosciences, San Jose, CA, USA)), endoglin (CD105-ImmunoglobulinG1-Allophycocyanin (MHCD10505, Thermo Fisher Scientific, Waltham, MA, USA)), homing cell adhesion molecule (CD44-ImmunoglobulinG2b-Fluorescein isothiocyanate (555478, BD Biosciences, San Jose, CA, USA)), integrin beta-1 (CD29-ImmunoglobulinG1-Allophycocyanin (1A-219-T100, Exbio, Praha, Czech Republic)), and macrophage protein, which binds lipopolysaccharide (CD14-ImmunoglobulinG2a-Allophycocyanin (301808, BioLegend, San Diego, CA, USA)), protein tyrosine phosphatase, receptor type C (CD45-ImmunoglobulinG2a-Fluorescein isothiocyanate (sc-70686, Santa Cruz Biotechnology, Dallas, TX, USA)) and thymocyte differentiation antigen 1 (CD90-ImmunoglobulinG1-Fluorescein isothiocyanate (328108, BioLegend, San Diego, CA, USA)). After staining with antibodies, cells were washed twice with PBS and 1% BSA and analyzed with a flow cytometer BD FACSAria II (BD Biosciences, San Jose, CA, USA).

### 4.3. Proliferation Assay

Cells (0.5 × 10^3^/well) were seeded into 96-well plates. The proliferation of healthy and pathological cells was measured using a CCK-8 reagent for 6 days according to the manufacturer’s recommendations (Dojindo, Kumamoto, Japan). Briefly, 5 µL of CCK8 was added to 150 µL of growth media, the 96-well plate was incubated at 37 °C for 3 h and absorption was measured at 450 nm using the SpectraMax i3 (Molecular Devices, San Jose, CA, USA) spectrophotometer. In parallel, resuspended cells were stained with trypan blue (Sigma Aldrich, St. Louis, MO, USA) and counted using a cell fast read counter (Biosigma, Cona, Italy).

### 4.4. Exposure of hMSCs to HDAC Inhibitors

Cells were seeded on a cell culture plate for the measurement of ATP level, mitochondria membrane potentials, HDAC activity, ROS and protein levels, and gene expression. When cells reached 80–90% confluence, the growth medium was changed to DMEM/F12 medium with 2% FBS and SAHA (1 µM) or mocetinostat (0.03 µM) or MC1568 (2 µM). Differentiation was induced for different time points to evaluate HDACi effect on hmMSCs. The concentrations of HDAC inhibitors were chosen so as not to affect cell viability during the 21 days of incubation.

### 4.5. Measurement of Histone Deacetylase Activity

Cells were lysed with HDAC activity buffers (50 mM Tris-HCl, pH = 7.5, 5% glycerol, 0.3%, Triton-100, 50 mM NaCl) and frozen at −80 °C for later use. Thawed samples were centrifuged at 15,000× *g* and +4 °C for 15 min and protein concentration was measured with Pierce™ Modified Lowry Protein Assay Kit (Thermo Fisher Scientific, Waltham, MA, USA). For each HDAC activity measurement, 50 µg of protein lysate was mixed with 30 µL of HDAC activity substrate Boc-Lys(Ac)-AMC (Sigma Aldrich, St. Louis, MO, USA) and the reaction was performed in the HDAC reaction buffer (50 mM Tris-HCl, pH = 8, 100 mM NaCl) at 30 °C for 30 min. The reaction was stopped with 10 mg/mL of trypsin and 25 µM of SAHA. Fluorescence was measured at 325/395 nm using a SpectraMax i3 spectrophotometer (Molecular Devices, San Jose, CA, USA).

### 4.6. Measurement of ATP Concentration

Cells (1 × 10^3^) were seeded in a white, clear-bottom 96-well plate. ATP measurement was performed using a ATPlite 1step Luminescence Assay kit and adding 100 µL of ATPlite 1step solution to each well as recommended by the manufacturer (PerkinElmer, Waltham, MA, USA). Measurement of chemiluminescence was performed using a SpectraMax i3 spectrophotometer (Molecular Devices, San Jose, CA, USA). ATPlite 1 step solution lyse cells measures ATP concentration, therefore, for the measurement of protein concentrations, the cells (1 × 10^3^) were in parallel seeded into a 96-well plate, lysed and measured with a Pierce™ Modified Lowry Protein Assay Kit (Thermo Fisher Scientific, Waltham, MA, USA). All ATP results were normalized to protein concentration.

### 4.7. Mitochondrial Membrane Potential (MMP) Measurement

Cells (1 × 10^3^) were seeded in a black, clear-bottom 96-well plate and were incubated with 2 µM of JC1 (Thermo Fisher Scientific, Waltham, MA, USA) in an IMDM medium at 37 °C for 30 min. MMP was evaluated by scanning each well with a SpectraMax i3 spectrophotometer and measuring red (high MMP) and green (low MMP) fluorescence. The more active MMP accumulate more JC-1 dye, which aggregates showing red fluorescence, while cells with lower MMP activity show green fluorescence of monomeric JC1. The ratio of red/green fluorescence shows the ratio of high/low MMP intensity.

### 4.8. Measurement of Oxidative Stress

Cells (7 × 10^3^) were seeded into a clear 12-well plate. Cultivated cells were trypsinized, washed with PBS and stained with a Muse^®^ Oxidative Stress Kit and analyzed using Muse^®^ Cell Analyzer according to the manufacturer’s recommendations (Merck Millipore, Burlington, MA, USA). Levels of ROS were measured both in control cells and cells exposed to 1 µM SAHA, 0.03 µM Mocetinostat and 2 µM MC1568 for 3 days.

### 4.9. Gene Expression Analysis

RNA was isolated with a GeneJET RNA Purification Kit (Thermo Fisher Scientific, Waltham, MA, USA) according to the manufacturer’s recommendations. Genomic DNA was digested with RNase-free DNase I (1 U/µL), also according to the manufacturers recommendations. A High-Capacity cDNA Reverse Transcription Kit was used for the cDNA synthesis. Real-time PCR was performed in triplicate using the 2× Maxima Probe qPCR Master Mix (Thermo Fisher Scientific, Waltham, MA, USA) on a AriaMx Real-Time PCR Machine (Agilent Technologies, Santa Clara, CA, USA). Gene expression was normalized to the housekeeping gene ACTB. A list of primers used to measure gene expression is shown in Table 1.

### 4.10. Western Blotting

Samples were lysed with a RIPA buffer (150 mM NaCl, 5 mM EDTA, 50 mM Tris, 1% NP-40, 0.5% sodium deoxycholate, 0.1% SDS, 1× tablet of protease inhibitors, 1 M NaF, 100 mM PMSF, and 200 mM Na_3_VO_4_). Protein concentration was determined with a Pierce™ Modified Lowry Protein Assay Kit (Thermo Fisher Scientific, Waltham, MA, USA), all samples were equilibrated with a 6× sample buffer with DTT (375 mM Tris-HCl pH 6.8, 6% SDS, 48% glycerol, 9% 2-mercaptoethanol, and 0.03% bromphenol blue), heated at 95 °C for 5 min to denature proteins. Protein electrophoresis was run on commercial Bolt 4–12% Bis-Tris Plus Gels (Thermo Fisher Scientific, Waltham, MA, USA). Proteins were transferred to a PVDF membrane under standard conditions at 20 V for 1 h. After the transfer, membranes were washed with TBST (Tris buffered saline with 20% Tween 20) and then blocked with 3% BSA in TBST at RT for 1 h. Primary antibodies were added to the membrane and incubated at 4 °C overnight. After the incubation with the primary antibody, the membrane was washed with TBST 3 × 10 min and probed with a HRP-conjugated secondary antibody at RT for 1 h and washed 4 × 5 min with TBST. SuperSignal West Pico chemiluminescent substrate (Thermo Fisher Scientific, Waltham, MA, USA) was added to detect probed proteins. Signal detection and fixation were performed using a Kodak developer and fixer.

### 4.11. Immunocytochemistry

Healthy and pathological hmMSCs were cultured on glass coverslips until they reached full confluence. To induce cardiac differentiation, we used Dulbecco’s modified Eagle’s medium (DMEM)/F12 supplemented with 2% fetal bovine serum (FBS) and 1 µM SAHA. The cells underwent differentiation for 3, 7, and 14 days, followed by fixation with 4% paraformaldehyde at RT for 15 min. Subsequently, they were permeabilized with 0.1% Triton-100 for 15 min and incubated with primary antibodies against alpha cardiac actin (GTX101876, GeneTex, Irvine, CA, USA), followed by secondary antibodies, conjugated with Alexa Fluor 488 (A16096, Thermo Fisher Scientific, Waltham, MA, USA). Additionally, the cell nuclei were stained with 1 µg/mL of 4′,6-diamidine-2′-phenylindole dihydrochloride (DAPI) for 2 min. Confocal images were captured using a Leica TCS SP8 confocal laser scanning microscope (Leica Microsystems, Wetzlar, Germany).

### 4.12. Statistics

Statistical analysis was conducted using Microsoft Excel 2016 (Microsoft Corporation, Redmont, WA, USA) and GraphPad Prism 6.01 (GraphPad Software, San Diego, CA, USA). The data are expressed as means ± standard deviation (mean ± SD) based on a minimum of 3–7 replicates from 2–3 independent experiments. For all experiments, cells from 2–3 healthy and dilated human myocardia were examined. The results are presented as data ± SD from no less than 3–5 repeats. Statistical significance was determined using the Student’s *t*-test indicating * *p* ≤ 0.05 and ** *p* ≤ 0.01.

## 5. Conclusions

In summary, our data suggest that myocardium-derived hmMSCs from DCM hearts exhibit larger sizes, poorer proliferation, and lower mitochondrial activity compared to healthy counterparts. Treatment with class I and II HDAC inhibitors (SAHA, mocetinostat, and MC1568) increased total ATP levels, improved mitochondrial activity, and slightly elevated ROS levels. Furthermore, these inhibitors stimulated cardiac differentiation-related transcription factors and structural proteins.

Given these findings, it is conceivable that HDAC inhibitors could play a role in treating DCM by enhancing the regenerative capacity of myocardium-derived hmMSCs. By improving mitochondrial function and ATP production, HDAC inhibitors may help restore the energy balance in DCM-affected hearts. Additionally, the stimulation of cardiac differentiation markers suggests that HDAC inhibitors could promote the repair and regeneration of damaged cardiac tissue. Although the slight increase in ROS levels warrants caution, this oxidative stress might also trigger protective signaling pathways that further contribute to cardiac repair mechanisms.

Overall, our study suggests that HDAC inhibitors warrant further investigation not only as anti-cancer compounds but also as potential therapeutics for DCM, aiming to regenerate the function of the dilated human heart. These compounds might offer dual benefits by simultaneously targeting pathological cellular mechanisms and enhancing the reparative processes essential for cardiac recovery.

## Figures and Tables

**Table 1:** List of gene expression primers.

Gene	Full Gene Name	Assay ID
*ACTB*	Actin beta	Hs01060665_g1
*ACTC1*	Actin Alpha Cardiac Muscle 1	Hs01109515_m1
*GATA4*	GATA Binding Protein 4	Hs00171403_m1
*HOPX*	HOP Homeobox	Hs04188695_m1
*MEF2C*	Myocyte Enhancer Factor 2C	Hs00231149_m1
*NKX2-5*	NK2 Homeobox 5	Hs00231763_m1
*TNNT2*	Troponin T2, Cardiac Type	Hs00943911_m1

## Data Availability

The data are not publicly available; their containing information could compromise the privacy of research participants.
